# Profiling of serum antibodies against human papillomavirus antigens in Korean women with cervical intraepithelial neoplasia and cervical cancer

**DOI:** 10.1002/cam4.1810

**Published:** 2018-10-23

**Authors:** Yingji Jin, Jae Woong Choi, Hyoung Jin Kim, Jamel Eddouzi, Seung Cheol Kim, Woong Ju, Yun Hwan Kim, Hong‐Jin Kim

**Affiliations:** ^1^ Laboratory of Virology, College of Pharmacy Chung‐Ang University Seoul South Korea; ^2^ Department of Obstetrics and Gynecology Ewha Womans University College of Medicine Seoul South Korea

**Keywords:** anti‐HPV antibody, cervical cancer, E6, E7 and L1, Sero‐epidemiology

## Abstract

Sero‐epidemiological studies of human papillomavirus (HPV) have been undertaken over the last two decades. In this study, the prevalences of nine serum antibodies (anti‐E6, E7 and L1 antibodies of HPV types 16, 18, and 58) were evaluated in normal (control) Korean women and women with cervical intraepithelial neoplasia (CIN) I, CIN II, CIN III, and cervical cancer. The frequencies of all types of anti‐HPV antibodies were higher in the CIN stages and cervical cancer than in normal women, and those of anti‐HPV16 E6 and E7, anti‐HPV18 E6 and E7, and anti‐HPV58 E7 antibodies were higher in the cervical cancer group than in the CIN stages. The frequencies of antibodies against HPV16, 18, and 58 E7 tended to increase with increasing severity of cervical lesions. However, there were few differences in the frequencies of antibodies against the L1 antigens of HPV16, 18 and 58 in cervical cancer versus CIN stages. The anti‐HPV antibodies were detected in 26.5% of normal, 46.3% of CIN I, 62.5% of CIN II, 51.6% of CIN III, and 75% of cancers when any of the nine antigens was used as a criterion. Correlations between HPV DNA positivity and seropositivity for anti‐HPV E6, E7, or L1 antibodies were found only in HPV16 DNA‐positive cervical cancers for anti‐HPV16 E6 and L1 antibodies. In addition, strong positive correlations in seropositivity were found between anti‐HPV16 E7 and anti‐HPV58 E7 antibodies, and between anti‐HPV18 E6 and anti‐HPV58 E6 antibodies. These findings should advance global profiling of the seroprevalences of antibodies against HPV antigens.

## 1 INTRODUCTION

Human papillomavirus (HPV) is a nonenveloped double‐stranded DNA virus found in 99.7% of patients with cervical cancer.^1^ More than 170 types of HPV have been identified, and they are subdivided into those with a high risk of causing cervical cancer (types 16, 18, 31, 33, 45, 52, and 58) and wart‐causing types (types 6 and 11) with a low risk of causing cervical cancer.^2^, ^3^ Invasive cervical cancer develops from cervical intraepithelial neoplasia (CIN), which comprises precancerous stages during which infection with a high‐risk HPV persists.^4^, ^5^, ^6^

Humoral immune responses to HPV L1, E6, and E7 antigens have been targets for studying the natural history of cervical carcinogenesis.^7^, ^8^ Various types of approaches such as enzyme‐linked immunosorbent assay (ELISA), proteome microarray, and radioimmune precipitation assay were applied for profiling the anti‐HPV antibody responses so far.^7^, ^8^, ^9^, ^10^, ^11^ It was suggested that the antibody responses to early and late HPV antigens occur at different times or phases of HPV pathogenesis.^9^ Meanwhile, the low sensitivities of anti‐HPV antibody‐based markers for detecting the cervical lesions were drawbacks indicated.^7^

There is evidence that geographical distribution affects HPV prevalences.^6^, ^12^ On the basis of HPV DNA analysis, HPV16 and 18 are the most common and second most common HPV types, respectively, in cervical cancer, and together are responsible for about 70% of cervical cancers worldwide.^13^ HPV58 is present in 3.3% of cervical cancers globally and is the fifth most common type (after HPVs 16, 18, 45, and 33) worldwide.^14^ However, it is the third most frequent type in cervical cancers in East Asia.^15^, ^16^ In fact, according to one report, it is actually the second most frequent type (after HPV16) in Korea, present in 16% of cervical cancers.^17^ This prevalence rate of HPV58 in Korea (16%) is significantly higher than that in Europe (1%).^18^

In this study, the seroprevalences of antibodies against nine types of HPV antigen (E6, E7, and L1 of HPV16, 18, and 58) were evaluated in Korean women with CIN I, CIN II, CIN III, and cervical cancer, and in normal controls.

## 2 MATERIALS AND METHODS

### 2.1 Study population

This study was carried out with the approval of the Ewha Womans University Mokdong Hospital Institutional Review Board (approval No. ECT 13‐15A‐28), and all samples were obtained from the same hospital. Samples were collected in a prospective manner after obtaining written informed consent from participants. A total of 249 serum samples were collected from women with normal cytology (n = 49), CIN I (n = 41), CIN II (n = 39), CIN III (n = 64), and cervical cancer (n = 56). Participants were screened by liquid‐based cytology prior to biopsies. Sera from the normal group were collected after examining hysterectomy specimens. Individuals with negative results in the examination of hematoxylin and eosin‐stained sections of hysterectomy specimens were classified as a normal group. Sera from the CIN I group were collected immediately after punch biopsy, and those from the CIN II and CIN III groups were collected before large loop excision of the transformation zone. Sera from cervical cancer patients were collected before surgery. Women over age 20 who have resulted with an abnormality in the cervix from the cytology examination and are designed for biopsy or surgery under suspicion of CINs or cervical cancer were included. Immunocompressed individuals (infection with human immunodeficiency virus, transplant operation, or immunocompressive medications) or individual who has record of another type of cancer was excluded.

Each cervical lesion was graded by hematoxylin and eosin review of sections cut from formalin‐fixed and paraffin‐embedded tissue blocks. Cervical cancer was graded according to the International Federation of Gynecology and Obstetrics (FIGO) staging system.

### 2.2 HPV DNA testing

HPV DNA was detected as described previously, with modifications.^19^ Samples for the HPV DNA testing were obtained from the liquid‐based cytology above. Polymerase chain reactions for HPV types 16, 18, and 58 were carried out in 20 µL volumes containing: 1× Go Taq reaction buffer (1.5 mM MgCl_2_), 10 mM dNTP Mix, 5U Go Taq DNA polymerase (Promega, USA), 0.1‐1.0 µM of each primer, and an extract of cervix cell lysate. Primers were as follows: for HPV16 E6 (target size 120 bp) forward 5′‐tcaaaagccactgtgtcctga‐3′ and reverse 5′‐cgtgttcttgatgatctgcaa‐3′; for HPV18 E6 (target size 202 bp) forward 5′‐cgacaggaacgactccaacga‐3′ and reverse 5′‐gctggtaaatgttgatgattaact‐3′; for HPV58 E7 (target size 109 bp) forward 5′‐cgaggatgaaataggcttgg‐3′ and reverse 5′‐acacaaacgaaccgtggtgc‐3′.

### 2.3 Enzyme‐linked immunosorbent assays

Enzyme‐linked immunosorbent assays (ELISAs) were used to detect serum antibodies against nine HPV antigens (E6, E7, and L1 of HPV types 16, 18, and 58). Glutathione S transferase‐fused E6 proteins were expressed in *Escherichia coli* and purified as described previously.^20^ 6× histidine‐tagged E7 proteins were expressed in *Escherichia coli* and purified by nickel‐nitrilotriacetic acid chromatography, and L1 proteins were expressed in *Saccharomyces cerevisiae* and purified as described previously.^21^ 96‐well ELISA plates (Greiner Bio‐One, Kremsmünster, Australia) were coated overnight with 100 ng of each viral protein at 4℃. The plates were blocked with 5% skim milk (Bioworld, Dublin, Ohio, USA) in phosphate‐buffered saline (PBS) containing 0.05% Tween 20 (PBS‐T) at room temperature (RT) for 2 hours. Then, 1:200 dilutions of sera in PBS‐T with 0.5% skim milk were incubated in the wells at RT for 2 hours Serum antibodies bound to the immobilized HPV antigens were detected with horseradish peroxidase‐conjugated goat anti‐human IgG antibody (Sigma, St. Louis, MO, USA, #A8667). The plates were washed three times with PBS‐T between reactions and five times before substrate reactions. Color was developed with *o*‐phenylenediamine dihydrochloride (Sigma) and measured at 492 nm with a Flexstation 3 multi‐mode microplate reader (Molecular Devices, San Jose, CA, USA).

### 2.4 Statistical analysis

Age differences between groups were analyzed by Student's *t* test. Differences in levels of antibodies between groups were evaluated using the Mann‐Whitney *U* test. Bonferroni corrections were performed for multiple comparisons. To identify seropositivity, cutoff values were set at the 95th percentile of the normal group. Differences between groups in the proportions of seropositivity were analyzed by Fisher's exact test with Bonferroni corrections for multiple comparisons. Chi‐square tests for trends were used to evaluate whether changes of antibody prevalence were significant. *P* < 0.05 was considered statistically significant in all tests. Relationships between antibody prevalences were analyzed with Spearman's rank correlation coefficient. All tests were conducted with GraphPad program version 6 (Graphpad software Inc, La Jolla, CA, USA).

## 3 RESULTS

### 3.1 Clinicopathological characteristics of normal, CIN I, CIN II, CIN III, and cervical cancer groups

The clinicopathological characteristics of cervical lesions are presented in Table 1. A total of 249 samples was collected from normal (n = 49), CIN I (n = 41), CIN II (n = 39), CIN III (n = 64), and cervical cancer (n = 56) groups. Mean ages of the normal, CIN I, CIN II, CIN III, and cervical cancer groups were 43, 44.2, 44.4, 39.7, and 50.0, respectively. The proportions of squamous cell carcinoma and adenocarcinoma among the cervical cancers were 71.4% and 23.2%, respectively, similar to those found generally (squamous cell carcinoma, 80%; adenocarcinoma, 20%).^22^ The frequencies of HPV DNAs in the cervical cancers were, in descending order, HPV16, 18, and 58, while in the CINs it was HPV16, 58, and 18 (Table 1). The frequencies of HPV16 and 18 DNAs showed increasing trends as the severity of cervical lesion increased (Table 1). No HPV DNA positives were found in normal group. HPV16, 18, and 58 DNA positives in CIN I and CIN II group were 14.6%, 7.3%, and 17.1% and 23.1%, 0%, and 12.8%, respectively, while those in CIN III and cervical cancer group were 45.3%, 9.4%, and 14.1% and 55.4%, 12.5%, and 5%, respectively.

**Table 1:** Clinicopathological characteristics of normal, CIN I, CIN II, CIN III, and cancer groups

	Normal (n = 49)	CIN I (n = 41)	CIN II (n = 39)	CIN III (n = 64)	Cancer (n = 56)	
Age, y (Mean ± SEM; Age range)^a^	43 ± 1.3; 26‐72	44.2 ± 2.0; 25‐74	44.4 ± 1.8; 28‐75	39.7 ± 1.6; 22‐74	50.0 ± 1.9; 28‐82	
HPV DNA positivity^b^						Trend^c^
16 DNA positivity	0%	14.6%	23.1%	45.3%	55.4%	*P* < 0.0001
18 DNA positivity	0%	7.3%	0%	9.4%	12.5%	*P* < 0.01
58 DNA positivity	0%	17.1%	12.8%	14.1%	5.0%	
Histology of cervical cancer (Punch biopsy)						Squamous cell carcinoma (n = 40; 71.4%) Endocervical adenocarcinoma (n = 13; 23.2%) Adenosquamous carcinoma (n = 2; 3.6%) Neuroendocrine carcinoma (n = 1; 1.8%)
Stage of cervical cancer^d^						Ia (n = 7; 12.5%)
						Ib (n = 32; 57.1%)
						IIa (n = 3; 5.4%)
						IIb (n = 11; 19.6%)
						IIIa (n = 1; 1.8%)
						IVa (n = 1; 1.8%)
						IVb (n = 1; 1.8%)

a Mean age of cancer group was higher than that of normal, CIN I, CIN II, or CIN III group. Comparison of age between groups was calculated by Student's *t* test: cancer vs normal, *P* < 0.01; cancer vs CIN I, *P* < 0.05; cancer vs CIN II, *P* < 0.05; cancer vs CIN III, *P* < 0.0001.

b HPV DNA positivity = number of HPV DNA presence sample × 100/total sample.

c Trends of HPV DNA positivity were analyzed by chi‐square for trend test.

d Stage of cervical cancer was classified by FIGO clinical staging system.

### 3.2 Comparison of seroprevalences of antibodies to nine HPV antigens in normal, CIN I, CIN II, CIN III, and cervical cancer groups

To compare the prevalences of serum antibodies against the nine HPV antigens in each cervical lesion group, antibody levels were measured by ELISA (Figure S1). The linearities and reproducibilities of the ELISAs were found to be excellent (Figure S2 and Table S1).

As shown in Table 2, the frequencies of the nine antibodies in all the cervical lesions (CIN I, CIN II, CIN III, and cancer) were generally higher than in the normal group. Antibodies against HPV16, 18, and 58 E7 antigens tended to increase in frequency with increasing stage of cervical lesion (*P* < 0.001 for each antibody; Table 2). Antibodies to HPV16 and HPV18 E6 also tended to be more common with increasing stage of cervical lesion, but the trends were weaker than for the antibodies against HPV16, 18, and 58 E7 (anti‐HPV16 E6, *P* < 0.01; anti‐HPV18 E6, *P* < 0.05; Table 2).

**Table 2:** Comparison of seroprevalence in the level of antibodies against HPV16/18/58 E6, E7, and L1 antigen in normal, CIN I, CIN II, CIN III, and cervical cancer groups

HPV type	Antigen	Normal (%)	CIN I (%)	CIN II (%)	CIN III (%)	Cancer (%)	Trend (Chi‐square for trend)
HPV 16	E6	4.08^a^	19.51	17.95	10.94	32.14	*P* < 0.01
	E7	4.08^b^	2.44^c^	12.82	7.81^d^	30.36	*P* < 0.0001
	L1	4.08	19.51	15.38	18.75	14.29	
HPV 18	E6	4.08	14.63	15.38	21.88	16.07	*P* < 0.05
	E7	4.08^e^	4.87	7.69	14.06	25	*P* < 0.001
	L1	4.08	9.76	12.82	12.50	12.50	
HPV 58	E6	4.08	14.63	12.82	18.75	14.29	
	E7	4.08^f^	4.87	10.26	7.81	26.79	*P* < 0.001
	L1	4.08	17.07	7.69	10.94	10.71	

Cutoff of the seroprevalence was set at the 95th percentile of normal group. Fisher's exac*t* test was used to compare the seroprevalence of the anti‐HPV antigen IgGs between groups. *P* value was adjusted by Bonferroni correction. Chi‐square for trend was used to evaluate seroprevalence trends of the IgGs with increasing stage of cervical lesions. *P* < 0.05 was considered statistically significant.

a Normal vs Cancer; *P* < 0.01 (Fisher's exact test with Bonferroni correction).

b Normal vs Cancer; *P* < 0.01 (Fisher's exact test with Bonferroni correction).

c CIN I vs Cancer; *P* < 0.01 (Fisher's exact test with Bonferroni correction).

d CIN III vs Cancer; *P* < 0.05 (Fisher's exact test with Bonferroni correction).

e Normal vs Cancer; *P* < 0.05 (Fisher's exact test with Bonferroni correction).

f Normal vs Cancer; *P* < 0.05 (Fisher's exact test with Bonferroni correction).

Serum anti‐HPV16 E6 and E7, anti‐HPV18 E6 and E7, and anti‐HPV58 E7 antibodies were more common in cervical cancer than in the CIN stages, whereas the frequencies of anti‐HPV16 L1, anti‐HPV18 L1, and anti‐HPV58 E6 and L1 antibodies differed little between cervical cancer and CIN stages (Table 2). Serum antibody frequencies against HPV16, 18, and 58 L1 increased from the CIN I stage and tended to be maintained up to the cancer stage itself (Table 2).

In conclusion, the seroprevalences of antibodies against E7 antigens (of HPV16, HPV18, and HPV58) appear to be the best indicators of the severity of cervical lesions.

### 3.3 Seroprevalences of antibodies against the nine HPV antigens as a function of HPV DNA prevalence

The question whether the seroprevalences of antibodies against HPV antigens are influenced by the status of HPV infection was investigated (Table 3). There were no major changes in antibody prevalence associated with HPV DNA prevalence. However, the frequencies of antibodies against HPV16 E6 and L1 were higher in HPV16 DNA‐positive cervical cancers than in HPV16 DNA‐negative ones. Thus, there is little correlation between anti‐HPV antibody seroprevalence and HPV DNA positivity, except in the case of HPV16 DNA.

**Table 3:** Seroprevalence of HPV DNA‐negative and HPV DNA‐positive individuals to nine types of HPV antigens

	Normal (n = 49) (%)	CIN I (n = 41) (%)			CIN II (n = 39) (%)			CIN III (n = 64) (%)			Cancer (n = 56) (%)		
HPV16		HPV16 DNA Negative (n = 35)	HPV16 DNA Positive (n = 6)	Total (n = 41)	HPV16 DNA Negative (n = 30)	HPV16 DNA Positive (n = 9)	Total (n = 39)	HPV16 DNA Negative (n = 35)	HPV16 DNA Positive (n = 29)	Total (n = 64)	HPV16 DNA Negative (n = 25)	HPV16 DNA Positive (n = 31)	Total (n = 56)
E6	4.1	22.9	0	19.5	20	11.1	18.0	11.4	10.3	10.9	12	**48.4** a	32.2
E7	4.1	2.9	0	2.4	16.7	0	12.8	8.57	6.9	7.8	32	29.0	30.4
L1	4.1	20.0	16.7	19.5	10	33.3	15.4	14.29	24.1	18.7	0	**25.8** b	14.3
HPV18		HPV18 DNA Negative (n = 38)	HPV18 DNA Positive (n = 3)	Total (n = 41)	HPV18 DNA Negative (n = 39)	HPV18 DNA Positive (n = 0)	Total (n = 39)	HPV18 DNA Negative (n = 58)	HPV18 DNA Positive (n = 6)	Total (n = 64)	HPV18 DNA Negative (n = 49)	HPV18 DNA Positive (n = 7)	Total (n = 56)
E6	4.1	15.8	0	14.6	15.4	0	15.4	20.7	33.3	21.8	18.4	0	16.1
E7	4.1	5.3	0	4.9	7.7	0	7.7	15.5	0	14.1	20.4	57.1	25
L1	4.1	10.5	0	9.8	12.8	0	12.8	13.8	0	12.5	14.3	0	12.5
HPV58		HPV58 DNA Negative (n = 34)	HPV58 DNA Positive (n = 7)	Total (n = 41)	HPV58 DNA Negative (n = 34)	HPV58 DNA Positive (n = 5)	Total (n = 39)	HPV58 DNA Negative (n = 55)	HPV58 DNA Positive (n = 9)	Total (n = 64)	HPV58 DNA Negative (n = 53)	HPV58 DNA Positive (n = 3)	Total (n = 56)
E6	4.1	14.7	14.3	14.6	14.7	0	12.82	18.2	22.2	18.75	13.21	33.3	14.3
E7	4.1	5.9	0	4.9	11.8	0	10.3	5.5	22.2	7.8	28.3	0	26.8
L1	4.1	20.6	0	17.1	5.8	20	7.5	10.9	11.1	11	11.3	0	10.7

Cutoff of seroprevalence was set at the 95th percentile of normal group. *P* value was analyzed by the Fisher's exact test to compare the seroprevalence of the IgGs between groups. Bolds are significant difference between DNA negative and positive.

a DNA positive vs DNA negative (anti‐HPV16 E6 antibody seroprevalence in cancer group): *P* < 0.01, Fisher's exact test was applied.

b DNA positive vs DNA negative (anti‐HPV16 L1 antibody seroprevalence in cancer group): *P* < 0.01, Fisher's exact test was applied.

### 3.4 Correlations between antibodies against the nine HPV antigens

The correlations between antibodies against the nine HPV antigen in normal, CIN I, CIN II, CIN III, and cervical cancer are presented in Table 4. *R* > 0.8 was considered a strong positive correlation. There were strong positive correlations between anti‐HPV16 E7 and anti‐HPV58 E7 antibodies, and between anti‐HPV18 E6 and anti‐HPV58 E6 antibodies in the normal, CIN III, and cancer groups (Table 4). A correlation between HPV18 E6 and HPV58 E6 was also found in the CIN I group. Thus, sera reactive with HPV16 E7 and HPV18 E6 tended to react with HPV58 E7 and HPV58 E6, respectively. We suggest that immune responses to HPV58 should be noted together with those to HPV16 and 18, when examining the development of cervical cancer.

**Table 4:** Correlation between the seroprevalences of antibodies against nine types of HPV antigens

		HPV16 E6	HPV16 E7	HPV16 L1	HPV18 E6	HPV18 E7	HPV18 L1	HPV58 E6	HPV58 E7	HPV58 L1
Normal	HPV16 E6		0.38	−0.28	0.28	0.11	0.09	0.28	0.40	−0.14
	HPV16 E7			−0.06	0.69	0.75	0.31	0.66	**0.81**	0.06
	HPV16 L1				−0.09	0.02	0.26	0.01	0.05	0.64
	HPV18 E6					0.55	0.27	**0.86**	0.66	0.00
	HPV18 E7						0.26	0.41	0.62	0.29
	HPV18 L1							0.34	0.39	0.35
	HPV58 E6								0.65	−0.02
	HPV58 E7									0.10
	HPV58 L1									
		HPV16 E6	HPV16 E7	HPV16 L1	HPV18 E6	HPV18 E7	HPV18 L1	HPV58 E6	HPV58 E7	HPV58 L1
CIN I	HPV16 E6		0.53	0.43	0.74	0.21	0.07	0.75	0.52	0.13
	HPV16 E7			0.47	0.61	0.58	0.14	0.63	0.66	0.11
	HPV16 L1				0.60	0.37	0.30	0.59	0.34	0.53
	HPV18 E6					0.30	0.18	**0.97**	0.63	0.36
	HPV18 E7						0.11	0.34	0.57	0.14
	HPV18 L1							0.16	−0.06	0.36
	HPV58 E6								0.64	0.24
	HPV58 E7									0.09
	HPV58 L1									
		HPV16 E6	HPV16 E7	HPV16 L1	HPV18 E6	HPV18 E7	HPV18 L1	HPV58 E6	HPV58 E7	HPV58 L1
CIN II	HPV16 E6		0.42	−0.07	0.46	0.25	0.12	0.45	0.36	0.03
	HPV16 E7			−0.21	0.44	0.73	0.35	0.55	0.66	−0.14
	HPV16 L1				−0.08	−0.13	−0.27	−0.12	0.04	0.57
	HPV18 E6					0.44	0.23	0.74	0.45	0.07
	HPV18 E7						0.19	0.56	0.54	0.05
	HPV18 L1							0.13	0.21	−0.28
	HPV58 E6								0.53	0.04
	HPV58 E7									0.12
	HPV58 L1									
		HPV16 E6	HPV16 E7	HPV16 L1	HPV18 E6	HPV18 E7	HPV18 L1	HPV58 E6	HPV58 E7	HPV58 L1
CIN III	HPV16 E6		0.49	−0.09	0.60	0.36	0.17	0.47	0.44	−0.04
	HPV16 E7			0.15	0.71	0.77	0.03	0.64	**0.81**	−0.03
	HPV16 L1				0.08	0.16	0.42	0.16	0.14	0.61
	HPV18 E6					0.40	0.13	**0.93**	0.74	0.00
	HPV18 E7						0.00	0.33	0.62	0.14
	HPV18 L1							0.13	0.03	0.23
	HPV58 E6								0.72	0.04
	HPV58 E7									0.12
	HPV58 L1									
		HPV16 E6	HPV16 E7	HPV16 L1	HPV18 E6	HPV18 E7	HPV18 L1	HPV58 E6	HPV58 E7	HPV58 L1
Cancer	HPV16 E6		0.30	0.11	0.05	0.12	−0.11	0.09	0.35	0.15
	HPV16 E7			0.01	0.30	0.58	−0.20	0.29	**0.80**	0.01
	HPV16 L1				−0.44	−0.07	−0.22	−0.48	0.04	0.55
	HPV18 E6					0.28	0.23	**0.92**	0.34	−0.21
	HPV18 E7						0.01	0.29	0.38	−0.09
	HPV18 L1							0.21	−0.08	−0.20
	HPV58 E6								0.32	−0.21
	HPV58 E7									−0.01
	HPV58 L1									

Correlations were analyzed by Spearman correlation. Bolds are cases when the *R* value is over 0.8 (high positive correlation) between two types of antibodies.

## 4 DISCUSSION

### 4.1 Practical use of ELISA‐based serology assay

In summary, our results imply that the seroprevalence of anti‐HPV E7 may be the best indicator of the severity of cervical lesions: The seroprevalences of antibodies against HPV16, 18, and 58 E7 appeared to be 30%, 25%, and 27% in cervical cancer group, respectively (Table 2). Meanwhile, current HPV DNA testing (Hybrid Capture II) provides over 90% sensitivity for detecting CIN II or worse (CIN II+).^23^ Therefore, it is thought that the sensitivities of the ELISA‐based serology assays are too low to consider practical use of primary screening of cervical lesions.

### 4.2 Correlation between expression of HPV antigens and prevalence of anti‐HPV antibodies

When HPV infection in the cervix persists, integration of HPV DNA into host chromosomes can occur, and integration rates increase with stage of cervical lesion.^24^, ^25^, ^26^ As a result, the expression of E6 and E7 proteins increases with stage of cervical lesion.^25^, ^26^ In this study, the prevalence of serum antibodies against HPV E6 and HPV E7 oncoproteins also increased with increasing stage of cervical lesion (Table 2). On the other hand, the expression of L1 (HPV capsid protein), unlike that of E6 and E7 antigens, is known to increase preferentially in CIN I stage and to decline with increasing severity of the cervical lesions,^26^ and we found, in agreement with this, that the prevalence of anti‐HPV L1 antibodies increased in CIN I and did not increase further with stage of cervical lesion (Table 2). Therefore, it seems that the limited expression of L1 protein in high‐grade CIN and cervical cancer limits the immune response to it.

### 4.3 Comparison with previous studies

Overall, as shown in Table S2, our findings for anti‐HPV E6, E7, and L1 antibodies are consistent with previous observations in terms of increased seroprevalence in cervical lesions.^27^, ^28^, ^29^, ^30^, ^31^, ^32^, ^33^, ^34^ Our results also show that the frequencies of antibodies against HPV E6 and E7 (in types 16 and 18) are higher in cervical cancer than in CIN stages. Similar trends in the seroprevalence of antibodies against HPV E6 and E7 antigen in Korean women have been reported previously.^35^

Meanwhile, we noted little difference in the frequencies of anti‐HPV16 or 18 L1 antibodies between CIN stages and cervical cancer proper (Table 2 and Table S2). In contrast, an increased prevalence of anti‐HPV16 L1 antibody in cervical cancer compared to CIN stages was found in Mexico.^36^

### 4.4 Correlation between HPV DNA prevalence and anti‐HPV antibody prevalence

It is thought that persistent infection with HPV can induce the production of antibodies that eventually lead to seroconversion.^37^ In this study, we enquired whether the presence of antibodies against antigens of a given type of HPV was associated with the retention of HPV DNA corresponding to that HPV type. Overall, the presence of HPV DNA was not associated with increased seroprevalence, except in the case of HPV16 DNA‐positive cervical cancer where higher anti‐HPV16 E6 and L1 antibody frequencies were found in HPV16 DNA‐positive cervical cancer than in HPV16 DNA‐negative cervical cancer (Table 3). Jean‐Damien et al^38^ similarly found that HPV16 DNA‐positive individuals had higher levels of anti‐HPV16 E6 and anti‐HPV16 L1 antibodies than HPV16 DNA‐negative individuals, and Chee et al^35^ found a similar prevalence trend in HPV16 DNA‐positive individuals. However, we did not observe the same correlation in HPV16 DNA‐positive CIN III (Table 3).

### 4.5 Correlation between antibodies against different HPV antigens

We found a strong positive correlation between anti‐HPV16 E7 and anti‐HPV58 E7 antibodies, and between anti‐HPV18 E6 and anti‐HPV58 E6 antibodies (Table 4). There is considerable amino acid sequence conservation within the same species of HPV, and this allows cross‐reactivity between anti‐HPV antibodies.^39^, ^40^ HPV 16 and 58 belong to the A9 species while HPV18 belongs to A7,^34^, ^35^ and there is also some amino acid sequence conservation between species. Previous research in Algeria and South India found a strong correlation between the seroprevalences of anti‐HPV16 E7 and anti‐HPV58 E7 antibodies.^38^ This is consistent with our findings, but a correlation between anti‐HPV18 E6 and anti‐HPV58 E6 antibodies was also noted,^38^ and these do not accord with our findings. This discrepancy may be due to differences in major histocompatibility complex (MHC) restriction of the production of antibodies in different ethnic groups.

### 4.6 HPV type 58: a unique causative of cervical cancer in East Asia

HPV58 DNA positivity is found in 3.3% of cervical cancers and is the fifth most frequent type worldwide.^14^ Meanwhile, HPV58 is the third most common type found in cervical cancers in East Asia, after HPV16 and HPV18.^15^, ^16^ High rates of HPV58 DNA prevalence of 26%, 8%, and 16% were reported in cancer patients in Shanghai (China), Japan, and Korea, respectively.^17^, ^41^, ^42^ Moreover, HPV58 was found in 17.2% of high‐grade CIN groups in East Asia.^43^ In our study cases, HPV58 was more common than HPV18 in CIN stages (Table 1). These findings indicate that HPV58 is an important causative agent of cervical cancer in East Asia. Our results confirm that Korean women not only have high levels of HPV58 DNA positivity but also high levels of antibody seropositivities to HPV58 antigens in CINs (Tables 1 and 2). Therefore, we suggest that HPV58 must be taken into account in the prevention, treatment, and diagnosis of cervical cancer in the East Asian region.

### 4.7 Limitations of this study

In the present study, only three types of HPVs (HPV16, 18, and 58) were considered to investigate the anti‐HPV antibody seroprevalences and HPV DNA positivities, and limited number of serum samples per group were used (n = 39‐64). Also, folding property or antigenicity of the HPV E6 or E7 produced in *E. coli *may be different from that of native antigen is a considerable factor in interpretation of the seroprevalences.

### 4.8 Use of antibodies against HPV antigens as biomarkers, and future directions for their use

Antibodies against nine types of HPV antigens were assessed as biomarkers for diagnosing cervical lesions, and we found that rates of detection of cervical lesions increased significantly when the seroprevalence factors were used in parallel (Table S3). When “any of nine antigens” was used as criterion, CIN II, CIN III, and cervical cancer were detected (CIN II, 61.5%; CIN III, 51.6%; cervical cancer, 75%). However, 26.5% of normal individuals also registered as seropositive (Table S3). Meanwhile, considerable parts of cervical lesions (CIN II, 38.5%; CIN III, 48.4%; cervical cancer, 25%) were not detectable when screening protocol using “any of nine antigens” was applied. It seems that the serology assays have limited usefulness as primary screening system. In the serial assay strategy, the detection rate was zero in all groups when the “all of nine antigens” combination strategy was used (Table S3). In the “all of E7 (HPVs16, 18, and 58)” combination strategy, no false positives were found in the normal or CIN I group, and seroprevalence displayed an increasing trend as the severity of cervical lesion increased. However, the rates of detection of CIN II, CIN III, and cervical cancer were too low for this strategy to be applied in practice (CIN II, 2.6%; CIN III, 3.6; cervical cancer, 12.5%).

All in all, the antibodies against E7 proteins appeared to have the highest potential of the anti‐HPV antibodies tested as markers for detecting cervical lesions (Table 2), and further studies are needed to identify peptide regions of the E7 proteins whose antibody levels reflect most accurately the severity of cervical lesions.

We believe that our results will contribute to global profiling of the prevalence of serum antibodies against HPV antigens. The accumulated results of such efforts are expected to provide the fundamental basis for monitoring and treating cervical lesions.

## CONFLICT OF INTEREST

None of the authors have conflict of interests to disclose.
