# Supplementary figures and images for: Profiling of serum antibodies against human papillomavirus antigens in Korean women with cervical intraepithelial neoplasia and cervical cancer
Source: Cancer Med. 2018 Oct 23;7(11):5655–64. doi: 10.1002/cam4.1810 (PMC6247075; doi:10.1002/cam4.1810)

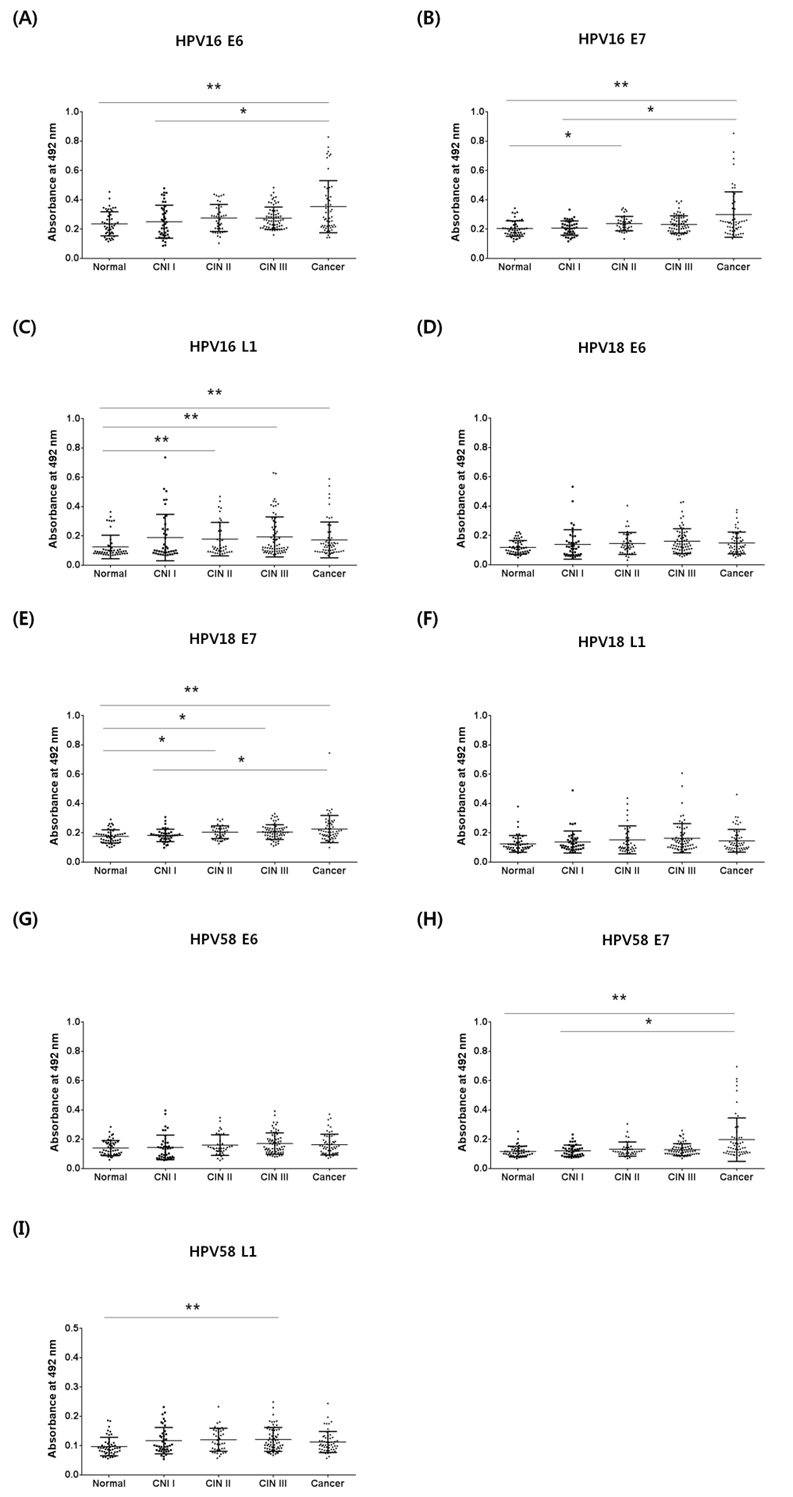

Supplement: Supplementary file 1 [file CAM4-7-5655-s001.tif]

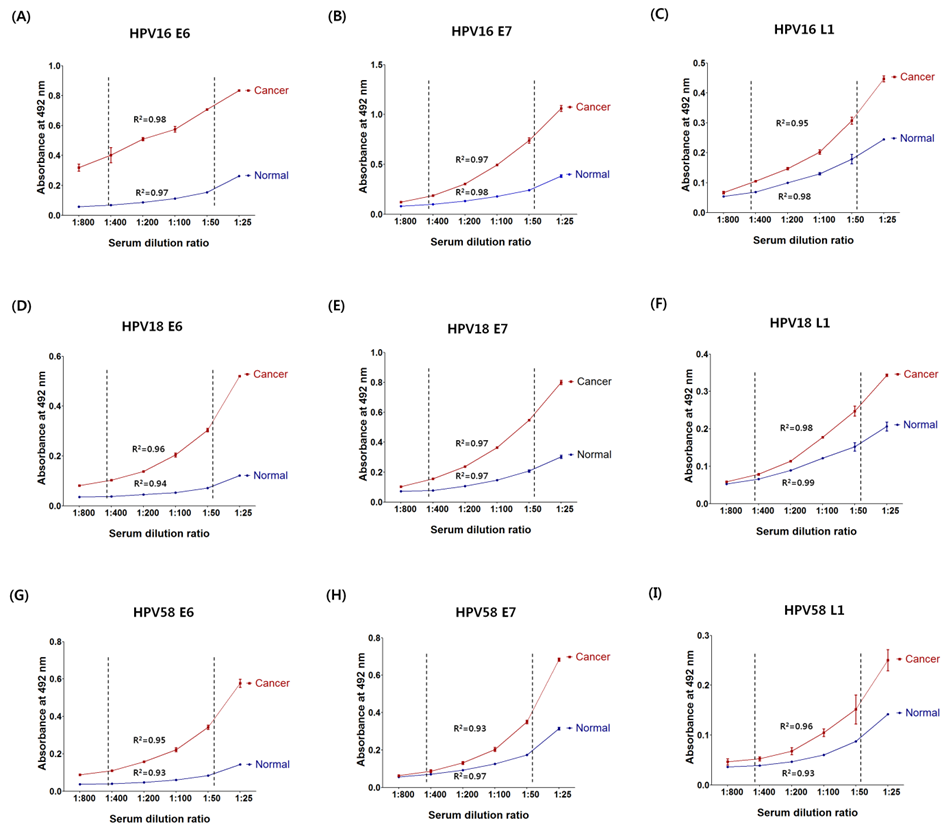

Supplement: Supplementary file 2 [file CAM4-7-5655-s002.tif]
