# Supplementary material for: Profiling of serum antibodies against human papillomavirus antigens in Korean women with cervical intraepithelial neoplasia and cervical cancer
Source: Cancer Med. 2018 Oct 23;7(11):5655–64. doi: 10.1002/cam4.1810 (PMC6247075; doi:10.1002/cam4.1810)
Supplement: Supplementary file 3 [file CAM4-7-5655-s003.docx]

Supplementary Table 1. Reproducibilities of ELISAs for evaluating levels of antibodies against nine types of HPV antigens. Ten sera samples were selected from low, medium or high level of antibody against the relevant HPV antigen, respectively. ELISAs were performed on the same plates (in triplicate) and the different plates (in triplicate) to evaluate intra- and inter-assay precision, respectively. OD, optical density; SD, standard deviation; CV, coefficient of variation.

|  |  | Intra-assay (Triplicate) | | | Inter-assay (Triplicate) | | |
| --- | --- | --- | --- | --- | --- | --- | --- |
|  |  | OD (Mean) | SD (Mean) | CV (Mean) | OD (Mean) | SD (Mean) | CV (Mean) |
| HPV16 E6 | Low (n=10) | 0.16 | 0.01 | 6.6% | 0.16 | 0.01 | 6.3% |
|  | Medium (n=10) | 0.24 | 0.02 | 6.1% | 0.24 | 0.01 | 6.0% |
|  | High (n=10) | 0.34 | 0.02 | 4.5% | 0.34 | 0.02 | 6.0% |
|  | Mean (n=30) |  |  | 5.7% |  |  | 6.1% |
| HPV18 E6 | Low (n=10) | 0.09 | 0.01 | 9.3% | 0.09 | 0.01 | 5.3% |
|  | Medium (n=10) | 0.19 | 0.02 | 8.1% | 0.20 | 0.01 | 4.8% |
|  | High (n=10) | 0.29 | 0.03 | 8.4% | 0.29 | 0.03 | 8.6% |
|  | Mean (n=30) |  |  | 8.6% |  |  | 6.3% |
| HPV58 E6 | Low (n=10) | 0.09 | 0.01 | 6.7% | 0.10 | 0.01 | 9.8% |
|  | Medium (n=10) | 0.18 | 0.01 | 5.2% | 0.20 | 0.02 | 8.2% |
|  | High (n=10) | 0.25 | 0.02 | 6.2% | 0.26 | 0.02 | 7.2% |
|  | Mean (n=30) |  |  | 6.0% |  |  | 8.4% |
| HPV16 E7 | Low (n=10) | 0.12 | 0.01 | 8.5% | 0.13 | 0.01 | 9.1% |
|  | Medium (n=10) | 0.26 | 0.02 | 5.7% | 0.25 | 0.02 | 6.8% |
|  | High (n=10) | 0.37 | 0.02 | 4.4% | 0.36 | 0.03 | 8.4% |
|  | Mean (n=30) |  |  | 6.2% |  |  | 8.1% |
| HPV18 E7 | Low (n=10) | 0.08 | 0.01 | 7.9% | 0.07 | 0.01 | 7.4% |
|  | Medium (n=10) | 0.20 | 0.02 | 8.7% | 0.22 | 0.02 | 8.8% |
|  | High (n=10) | 0.31 | 0.03 | 8.0% | 0.31 | 0.02 | 6.8% |
|  | Mean (n=30) |  |  | 8.2% |  |  | 7.7% |
| HPV58 E7 | Low (n=10) | 0.07 | 0.01 | 8.8% | 0.08 | 0.01 | 8.5% |
|  | Medium (n=10) | 0.17 | 0.02 | 8.5% | 0.17 | 0.01 | 7.7% |
|  | High (n=10) | 0.27 | 0.02 | 5.9% | 0.28 | 0.02 | 6.6% |
|  | Mean (n=30) |  |  | 7.7% |  |  | 7.6% |
| HPV16 L1 | Low (n=10) | 0.10 | 0.01 | 6.5% | 0.10 | 0.01 | 7.6% |
|  | Medium (n=10) | 0.23 | 0.01 | 6.1% | 0.22 | 0.01 | 4.5% |
|  | High (n=10) | 0.34 | 0.02 | 5.4% | 0.36 | 0.02 | 4.9% |
|  | Mean (n=30) |  |  | 6.0% |  |  | 5.7% |
| HPV18 L1 | Low (n=10) | 0.13 | 0.01 | 9.2% | 0.14 | 0.01 | 8.6% |
|  | Medium (n=10) | 0.20 | 0.02 | 8.3% | 0.23 | 0.02 | 9.4% |
|  | High (n=10) | 0.31 | 0.03 | 10.0% | 0.33 | 0.03 | 9.2% |
|  | Mean (n=30) |  |  | 9.2% |  |  | 9.1% |
| HPV58 L1 | Low (n=10) | 0.07 | 0.01 | 9.5% | 0.08 | 0.01 | 9.1% |
|  | Medium (n=10) | 0.15 | 0.01 | 8.5% | 0.14 | 0.01 | 8.8% |
|  | High (n=10) | 0.20 | 0.02 | 9.8% | 0.19 | 0.01 | 7.5% |
|  | Mean (n=30) |  |  | 9.3% |  |  | 8.5% |

Supplementary Table 2. Comparison of the seroprevalences of anti-HPV antibodies between this study and previous studies.

| Antibody | Normal  % | CIN I  % | CIN II  % | CIN III  % | Cancer  % | Cut-off (Based on normal value) | Assay | Region | Reference^a^ |
| --- | --- | --- | --- | --- | --- | --- | --- | --- | --- |
| 16 E6 | 4.1% | 19.5% | 18.0% | 10.9% | 32.2% | 95^th^ of normal | ELISA | Korea | Our study |
|  | 1% | N/A | | | 32% | 5SD | ELISA | Algeria and India | 1 |
|  | 6.0% | N/A | | | 54.1% | Mean+3SD | Radioimmunoprecipitation | Brazil | 2 |
|  | 0% | 7% | | | 51% | Mean+3SD | Radioimmunoprecipitation assay | Korea | 3 |
|  | 0% | N/A | | | 25.6% | Mean+3SD | ELISA | Mexico | 4 |
|  | 0.5% | N/A | | | 30.4% | Mean+3SD | ELISA | Tanzania | 4 |
|  | 4.0% | 4.2% | | | 36.8% | Mean+3SD | Radioimmunoprecipitation assay | Korea | 5 |
| 16 E7 | 4.1% | 2.4% | 12.8% | 7.8% | 30.4% | 95^th^ of normal | ELISA | Korea | Our study |
|  | 38.7% | 32.7% | 33.96% | | 80% | Mean+2SD | ELISA | Mexico | 6 |
|  | 3% | N/A | | | 28% | 5SD | ELISA | Algeria and India | 1 |
|  | 4.6% | N/A | | | 30.4% | Mean+3SD | Radioimmunoprecipitation | Brazil | 2 |
|  | 0% | N/A | | | 16.9% | Mean+3SD | ELISA | Tanzania | 4 |
|  | 7% | 11% | | | 33% | Mean+3SD | Radioimmunoprecipitation assay | Korea | 3 |
|  | 0% | N/A | | | 18.6% | Mean+3SD | ELISA | Mexico | 4 |
|  | 2.0% | 4.2% | | | 19.1% | Mean+3SD | Radioimmunoprecipitation assay | Korea | 5 |
|  | 5% | N/A | | | 18% | Staining intensity | Western immunoblot assay | Japan | 7 |
| 16 L1 | 4.1% | 19.5% | 15.4% | 18.7% | 14.3% | 95^th^ of normal | ELISA | Korea | Our study |
|  | 9.3% | 7.9% | 5.7% | | 40% | Mean+2SD | ELISA | Mexico | 6 |
|  | 4% | N/A | | | 19% | 5SD | ELISA | Algeria and India | 1 |
|  | 24.4% | N/A | | | 47.4% | A>0.183 | ELISA | Brazil | 2 |
|  | 17.7% | N/A | | | 55.1% | Mean+3SD | ELISA | Colombia | 8 |
|  | 20.8% | N/A | 59.2% | | 67.4% | Mean+2SD | ELISA | Korea | 9 |
|  | 16% | 27% | | | N/A | 0.319 | ELISA | Japan | 10 |
|  | 4% | N/A | | | 23% | Mean+2SD | ELISA | Japan | 11 |
|  | 2% | 18% | | | N/A | Mean+2SD | ELISA | Japan | 11 |
| 18 E6 | 4.1% | 14.6% | 15.4% | 21.8% | 16.1% | 95^th^ of normal | ELISA | Korea | Our study |
|  | 3% | N/A | | | 40% | 5SD | ELISA | Algeria and India | 1 |
|  | 0% | N/A | | | 7.8% | Mean+3SD | ELISA | Mexico | 4 |
|  | 0% | N/A | | | 13.2% | Mean+3SD | ELISA | Tanzania | 4 |
| 18 E7 | 4.1% | 4.9% | 7.7% | 14.1% | 25% | 95^th^ of normal | ELISA | Korea | Our study |
|  | 1% | N/A | | | 13% | 5SD | ELISA | Algeria and India | 1 |
|  | 0% | N/A | | | 15.5% | Mean+3SD | ELISA | Mexico | 4 |
|  | 0.5% | N/A | | | 14.2% | Mean+3SD | ELISA | Tanzania | 4 |
| 18 L1 | 4.1% | 9.8% | 12.8% | 12.5% | 12.5% | 95^th^ of normal | ELISA | Korea | Our study |
|  | 5% | N/A | | | 10% | 5SD | ELISA | Algeria and India | 1 |
|  | 20.4% | N/A | | | 42.2% | Mean+3SD | ELISA | Colombia | 8 |
|  | 1% | N/A | | | 13% | Mean+2SD | ELISA | Japan | 11 |
|  | 1% | 23% | | | N/A | Mean+2SD | ELISA | Japan | 11 |
| 58 E6 | 4.1% | 14.6% | 12.8% | 18.8% | 14.3% | 95^th^ of normal | ELISA | Korea | Our study |
| 58 E7 | 4.1% | 4.9% | 10.3% | 7.8% | 26.8% | 95^th^ of normal | ELISA | Korea | Our study |
| 58 L1 | 4.1% | 17.1% | 7.5% | 11.0% | 10.7% | 95^th^ of normal | ELISA | Korea | Our study |
|  | 20.1% | N/A | | | 34.7% | Mean+3SD | ELISA | Colombia | 8 |
|  | 11% | 31% | | | N/A | 0.304 | ELISA | Japan | 10 |
|  | 11% | N/A | | | 28% | Mean+2SD | ELISA | Japan | 11 |
|  | 10% | 13% | | |  | Mean+2SD | ELISA | Japan | 11 |

^a^ References

1. Combes JD, Pawlita M, Waterboer T et al. Antibodies against high-risk human papillomavirus proteins as markers for invasive cervical cancer. *Int J Cancer* 2014;**135**: 2453-61.

2. Sun YP, Eluf-Neto J, Bosch FX et al. Serum antibodies to human papillomavirus 16 proteins in women from Brazil with invasive cervical carcinoma. *Cancer Epidem Biomar* 1999;**8**: 935-40.

3. Park JS, Park DC, Kim CJ et al. HPV-16-related proteins as the serologic markers in cervical neoplasia. *Gynecol Oncol* 1998;**69**: 47-55.

4. Meschede W, Zumbach K, Braspenning J et al. Antibodies against early proteins of human papillomaviruses as diagnostic markers for invasive cervical cancer. *J Clin Microbiol* 1998;**36**: 475-80.

5. Chee YH, Namkoong SE, Kim DH et al. Immunologic diagnosis and monitoring of cervical cancers using in vitro translated HPV proteins. *Gynecol Oncol* 1995;**57**: 226-31.

6. Salazar-Pina DA, Pedroza-Saavedra A, Cruz-Valdez A et al. Validation of Serological Antibody Profiles Against Human Papillomavirus Type 16 Antigens as Markers for Early Detection of Cervical Cancer. *Medicine* 2016;**95**.

7. Paez CG, Yaegashi N, Sato S et al. Prevalence of Serum Igg Antibodies for the E7 and L2 Proteins of Human Papillomavirus Type-16 in Cervical-Cancer Patients and Controls. *Tohoku J Exp Med* 1993;**170**: 113-21.

8. Combita AL, Bravo MM, Touze A et al. Serologic response to human oncogenic papillomavirus types 16, 18, 31, 33, 39, 58 and 59 virus-like particles in colombian women with invasive cervical cancer. *Int J Cancer* 2002;**97**: 796-803.

9. Jeong NH, Lee NW, Woo MK et al. Serologic response to human papillomavirus type 16 virus-like particles in Korean women with cervical precancerous and cancerous lesions. *Arch Pharm Res* 2009;**32**: 383-9.

10. Matsumoto K, Yoshikawa H, Yasugi T et al. IgG antibodies to human papillomavirus 16, 52, 58, and 6 L1 capsids: Case-control study of cervical intraepithelial neoplasia in Japan. *J Med Virol* 2003;**69**: 441-6.

11. Matsumoto K, Yoshikawa H, Taketani Y et al. Antibodies to human papillomavirus 16, 18, 58, and 6b major capsid proteins among Japanese females. *Jpn J Cancer Res* 1997;**88**: 369-75.

Supplementary Table 3. Seroprevalence of antibodies in cervical lesions by combining seroprevalence factors with parallel or serial assay. Chi-square for trend test was used to evaluate seroprevalence trends of IgGs with increasing stage of cervical lesions. P<0.05 was considered statistically significant (*p*<0.05: *; *p*<0.01: **; *p*<0.001: ***, *p*<0.0001: ****).

|  |  | Normal | CIN I | CIN II | CIN III | Cancer | Trend |
| --- | --- | --- | --- | --- | --- | --- | --- |
| **Parallel assay** | Any of HPV16 (HPV16 E6/E7/L1) | 12.2% | 29.3% | 33.3% | 34.4% | 48.2% | *** |
|  | Any of HPV18 (HPV18 E6/E7/L1) | 6.1% | 24.4% | 30.8% | 35.9% | 44.6% | **** |
|  | Any of HPV58 (HPV58 E6/E7/L1) | 10.2% | 22.0% | 28.2% | 31.3% | 42.9% | *** |
|  | Any of E6 (HPV16/18/58 E6) | 12.2% | 24.4% | 30.8% | 31.3% | 42.9% | *** |
|  | Any of E7 (HPV16/18/58 E7) | 8.2% | 7.3% | 20.5% | 15.6% | 41.1% | **** |
|  | Any of L1 (HPV16/18/58 L1) | 10.2% | 31.7% | 30.8% | 31.3% | 32.1% | * |
|  | **Any of 9 antigens**  **[HPV16/18/58 (E6, E7 and L1)]** | **26.5%** | **46.3%** | **61.5%** | **51.6%** | **75%** | ******** |
| **Serial assay** | All of HPV16 (HPV16 E6/E7/L1) | 0% | 0% | 0% | 0% | 5.4% |  |
|  | All of HPV18 (HPV18 E6/E7/L1) | 0% | 0% | 0% | 1.6% | 0% |  |
|  | All of HPV58 (HPV58 E6/E7/L1) | 0% | 2.4% | 0% | 0% | 1.8% |  |
|  | All of E6 (HPV16/18/58 E6) | 0% | 9.8% | 5.1% | 4.7% | 5.4% |  |
|  | All of E7 (HPV16/18/58 E7) | 0% | 0% | 2.6% | 3.1% | 12.5% | ** |
|  | All of L1 (HPV16/18/58 L1) | 0% | 2.4% | 0% | 1.6% | 1.8% |  |
|  | **All of 9 antigens**  **[HPV16/18/58 (E6, E7 and L1)]** | **0%** | **0%** | **0%** | **0%** | **0%** |  |

Supplementary Figure 1. Comparison of levels of antibodies against the E6, E7 and L1 antigens of HPV16, HPV18 and HPV58 in normal, CIN I, CIN II, CIN III and cervical cancer groups. Details of the detection of antibodies against the relevant HPV antigens by ELISA are given in Materials and Methods. Center lines are mean values, and error bars show ranges of SD values. Normal, n=49; CIN I, n=41; CIN II, n=39; CIN III, n=64; Cervical cancer, n=56. *P*-values were calculated by Mann-Whitney-U test. Bonferroni corrections were made, and *p*<0.05 was considered statistically significant (*p*<0.05: *; *p*<0.01: **). A, B, C, D, E, F, G, H and I present the results for antibodies against HPV16 E6, HPV16 E7, HPV16 L1, HPV18 E6, HPV18 E7, HPV18 L1, HPV58 E6, HPV58 E7 and HPV58 L1, respectively.

Supplementary Figure 2. Evaluation of the linearity of ELISAs. Serum mixtures were prepared using 10 individual sera each from the normal or cervical cancer group to monitor linearity. 96-well ELISA plates were coated with the relevant HPV antigens and blocked with 5% skim milk in PBST. Then the serum mixtures were diluted serially (two-fold) from 1:25 to 1:800 and incubated on the plates at room temperature for 2 hrs. The subsequent steps were as described in Materials and Methods. The data are means ± SDs of two independent assays. Blue lines indicate the normal group, red lines the cancer group. R^2^ values were used to assess the linearity of each ELISA. A, B, C, D, E, F, G, H and I are the results for antibodies against HPV16 E6, HPV16 E7, HPV16 L1, HPV18 E6, HPV18 E7, HPV18 L1, HPV58 E6, HPV58 E7 and HPV58 L1, respectively.
